# Classical and novel properties of Holliday junction resolvase SynRuvC from *Synechocystis* sp. PCC6803

**DOI:** 10.3389/fmicb.2024.1362880

**Published:** 2024-04-18

**Authors:** Yanchao Gu, Yantao Yang, Chunhua Kou, Ying Peng, Wenguang Yang, Jiayu Zhang, Han Jin, Xiaoru Han, Yao Wang, Xihui Shen

**Affiliations:** ^1^State Key Laboratory for Crop Stress Resistance and High-Efficiency Production, Shaanxi Key Laboratory of Agricultural and Environmental Microbiology, College of Life Sciences, Northwest A&F University, Yangling, Shaanxi, China; ^2^Suzhou XinBio Co., Ltd., Suzhou, Jiangsu, China

**Keywords:** RuvC, Holliday junction resolvases, flap endonuclease (FEN), replication fork intermediate (Ref-I) cleavage activity, *Synechocystis* sp. PCC6803

## Abstract

Cyanobacteria, which have a photoautotrophic lifestyle, are threatened by ultraviolet solar rays and the reactive oxygen species generated during photosynthesis. They can adapt to environmental conditions primarily because of their DNA damage response and repair mechanisms, notably an efficient homologous recombination repair system. However, research on double-strand break (DSB) repair pathways, including the Holliday junction (HJ) resolution process, in *Synechocystis* sp. PCC6803 is limited. Here, we report that SynRuvC from cyanobacteria *Synechocystis* sp. PCC6803 has classical HJ resolution activity. We investigated the structural specificity, sequence preference, and biochemical properties of SynRuvC. SynRuvC strongly preferred Mn^2+^ as a cofactor, and its cleavage site predominantly resides within the 5′-TG↓(G/A)-3′ sequence. Interestingly, novel flap endonuclease and replication fork intermediate cleavage activities of SynRuvC were also determined, which distinguish it from other reported RuvCs. To explore the effect of SynRuvC on cell viability, we constructed a knockdown mutant and an overexpression strain of *Synechocystis* sp. PCC6803 (*synruvC^KD^* and *synruvC^OE^*) and assessed their survival under a variety of conditions. Knockdown of *synruvC* increased the sensitivity of cells to MMS, HU, and H_2_O_2_. The findings suggest that a novel RuvC family HJ resolvase SynRuvC is important in a variety of DNA repair processes and stress resistance in *Synechocystis* sp. PCC6803.

## Introduction

The efficient repair of double-strand breaks (DSBs) in DNA is critical to preserving genome stability and ensuring cell viability. Homologous recombination (HR) repairs DSBs and promotes genetic diversity (West, 2003; Mehta and Haber, 2014). During HR, helicases and nucleases break the DNA strand into single-stranded DNA tails with 3′-extensions. HR involves homology searching for template DNA, invasion of DNA strands, repair of DNA, and the migration and resolution of the joint (Wright et al., 2018).

At the end of HR, two DNA duplexes will form a four-way junction structure of DNA intermediates known as a Holliday junction (HJ), following homologous pairing and strand exchange (Holliday, 2007). HJ and similar cruciform structures can also form during the processes of post-replication repair and replication fork reversal. Replication fork reversal is a mechanism of rescuing stalled replication forks during DNA replication (Michel et al., 2007). HJ are resolved by HJ resolvases, a group of DNA structure-specific endonucleases that cleave the two crossing strands at the junction (Lilley and White, 2001; Matos and West, 2014). Based on their similar functional properties, HJ resolvases have been discovered in a wide range of species (Komori et al., 2000; Yang et al., 2012; Bauknecht and Kobbe, 2014; Carreira et al., 2022; Sun et al., 2022). Bioinformatic investigations of the evolutionary relationships among HJ resolvases have suggested that they independently originated from four distinct domains—endonuclease, endonuclease VII-colicin E, RusA, and RNase H-like domains (Aravind et al., 2000).

RuvC, a member of the RNase H-like subfamily, is principally responsible for processing HJs in most bacterial taxa (Dunderdale et al., 1994). To resolve HJs, the dimeric endonuclease RuvC introduces two symmetric 5′-phosphorylated cuts near the junction (Dunderdale et al., 1994). This produces two distinct recombinant dsDNA, which can be repaired by DNA ligases (Iwasaki et al., 1991; Bennett et al., 1993). In *Deinococcus radiodurans* (DrRuvC), *Escherichia coli* RuvC (EcRuvC), and *Pseudomonas aeruginosa* RuvC (PaRuvC), RuvCs function as homodimers, exhibiting HJ-specific endonuclease activity and displaying sequence preferences: DrRuvC (5′-(G/C)TC↓(G/C)-3′), EcRuvC (5′-(A/T)TT↓(G/C)-3′), and PaRuvC (TTC) (Shah et al., 1994; Hu et al., 2020; Sun et al., 2022). *ruvC* knockout strains of *E. coli and Helicobacter pylori* are viable but have reduced DNA repair efficiency (Mandal et al., 1993; Loughlin et al., 2003). Pure homozygous *ruvC* knockout strains of several bacterial taxa, such as *D. radiodurans*, could not be obtained as the vital role of RuvC in recombination (Sun et al., 2022).

Cyanobacteria have inhabited the Earth for approximately 3.5 billion years ago and adapted to environmental conditions by means of a variety of morphological and physiological transformations (Brock, 1973). Because of their photoautotrophic lifestyle, cyanobacteria are threatened by ultraviolet (UV) rays solar and the reactive oxygen species (ROS) produced during photosynthesis (Cassier-Chauvat and Chauvat, 2014). As a result, *Synechocystis* sp. PCC6803 and *Synechococcus elongatus* sp. PCC7942 have greater resistance to UV radiation than the non-photosynthetic bacterium *E. coli*, which has efficient DNA repair mechanisms (Baharoglu and Mazel, 2014). Moreover, *Synechocystis* sp. PCC6803 has greater resistance to gamma rays than *Synechococcus elongatus* sp. PCC7942 and *E. coli* (Domain et al., 2004). Research on DSB repair pathways, including the HJ resolution, in *Synechocystis* sp. PCC6803 is lacking. The genome of *Synechocystis* sp. PCC6803 contains a homolog of *ruvC* (*sll0896*), which encodes the putative HJ resolvase SynRuvC. However, the biological functions and enzymatic properties of SynRuvC are unknown.

Here, we report that SynRuvC has classical HJ resolvase activity, as well as novel flap endonuclease (FEN) and replication fork intermediate (Ref-I) cleavage activities. Notably, SynRuvC showed a preference for Mn^2+^ as a cofactor. The cleavage site of SynRuvC is primarily situated within the 5′-TG↓(G/A)-3′ sequence. Additionally, SynRuvC is esential for the survival of *Synechocystis* sp. PCC6803. The enzymatic properties and functions of SynRuvC were characterized. The findings provide insight into the DSB repair mechanisms of *Synechocystis* sp. PCC6803 and provide a theoretical basis for its stress resistance.

## Materials and methods

### Phylogenetic analysis

Amino acid sequences of RuvC proteins from 35 species were obtained from the NCBI website.^1^ A phylogenetic tree was constructed using the complete amino acid sequences of 35 proteins from SynRuvC and other species. Protein amino acid sequences were aligned using the ClustalX1.81 program and phylogenetic trees were constructed by neighbour-joining (NJ) method using MEGA6 software.

### DNA substrates

All oligonucleotides utilized in this work were bought from Sangon (Shanghai, China), and the sequences are given in Supplementary Table 1. For all DNA substrates, one strand of DNA is fluorescently labeled at the 5′ end with 6-carboxyfluorescein (FAM). DNA annealing was performed by mixing a 1:2 ratio of labeled and unlabeled oligonucleotides in the annealing buffer (20 mM Tris-HCl [pH 8.0], 50 mM NaCl), boiled in a water bath, and cooled to room temperature overnight (Qin et al., 2022).

### Protein expression and purification

The WT and other mutant variants of SynRuvC were expressed and purified in a similar way. In brief, the gene encoding SynRuvC (KEGG ID: *sll0896*) was amplified by PCR (polymerase chain reaction) and cloned into the expression vector pET15b with an N-terminal His-SUMO (small ubiquitin-like modifier) tag. The constructed recombinant vector was transformed into BL21(DE3) competent cells and grown on LB plate with 100 mg/L ampicillin. Transformed BL21(DE3) clones were cultured to an optical density of 0.4 to 0.6 at 600 nm in an LB medium containing 100 mg/L ampicillin at 37°C. IPTG (isopropyl β-D-1-thiogalactopyranoside) at a final concentration of 0.5 mM was used to stimulate protein expression at 18 °C for 18 h.

After harvesting, cells were resuspended in lysis buffer (20 mM Tris-HCl [pH 8.0], 0.5 mM NaCl, 10% [vol/vol] glycerol, and 5 mM imidazole), disrupted by sonication, and centrifuged at 6200 g at 4°C for 60 min. Additionally, the supernatants were injected into a Ni-NTA, desalting, and ion exchange (HiTrap Q HP column, GE Healthcare) columns utilizing AKTA pure (GE Healthcare). SDS-PAGE was used to confirm each fraction before being concentrated, aliquoted, and kept at −80°C.

### DNA binding assay

DNA binding assay was performed according to a previously reported approach (Sun et al., 2022), with some modifications. 50 nM 5′-FAM labeled substrates were mixed with various concentrations of SynRuvC in a 10 μL reaction buffer containing 20 mM HEPES (pH 7.5), 50 mM NaCl, 1 mM DTT, 10% (vol/vol) DMSO, and 1% (vol/vol) glycerol. Binding reactions were incubated at 37°C for 60 min, and the products were resolved by 5% native-PAGE (native polyacrylamide gels) in 1× Tris-Acetate-EDTA buffer. Gels were scanned by a fluorescent imaging system (Tanon 5200Multi, China).

### DNA cleavage assay

50 nM 5′-FAM labeled substrates were mixed with various concentrations of SynRuvC in a 10 μL reaction buffer containing 20 mM Tris-HCl (pH 7.5), 50 mM NaCl, 1 mM DTT, 10% (vol/vol) DMSO, 10 mM Mn^2+^, and 1% (vol/vol) glycerol. The reactions were incubated at 37°C for 60 min before being stopped with a solution containing proteinase K, SDS, and EDTA (Qin et al., 2022). All products were resolved by 10% native-PAGE in 1× Tris-Acetate-EDTA buffer. Gels were scanned by a fluorescent imaging system (Tanon 5200Multi, China). For the metal ion dependence experiment, MnCl_2_ was replaced with various concentrations of MgCl_2_, ZnCl_2_, CaCl_2_, CuCl_2_, CoCl_2_, and NiCl_2_. For the temperature effects experiment, reactions were carried out at the specified temperatures for 60 min. For the heat stability experiment, SynRuvC alone was pre-incubated at various temperature for 15 min in the reaction buffer and further incubated with DNA substrates at 37°C for 60 min. For the pH dependence experiment, Tris-HCl pH 7.5 was replaced by different pH of Tris-HCl. For the effect of salt concentrations experiment, 50 mM NaCl was replaced with various concentrations of KCl and NaCl. For the time-course analysis, reactions were carried out at 37 °C for different min.

### Determination of cleavage sites

For the determination of cleavage sites, each DNA substrates (50 nM) with a uniquely 5′-strand FAM-labeled arm were incubated with SynRuvC (2 μM) at 37°C for 60 min in reaction buffer, as mentioned above. The reaction was stopped by a solution containing proteinase K, SDS, and EDTA, and the products were resolved by 15 or 18% denaturing PAGE (containing 8 M urea) in 0.5× TBE (Tris-borate-EDTA) buffer. Gels were scanned by a fluorescent imaging system (Tanon 5200Multi, China). GA ladders of each labeled oligonucleotide produced by the Maxam-Gilbert technique were put alongside to serve as markers.

### Ligation reaction

5′-strand FAM-labeled 4Jhs (50 nM) was incubated with or without SynRuvC (2 μM) at 37°C for 60 min in reaction buffer (200 μL) and then stopped by phenol extraction (Komori et al., 2000). These products were precipitated using ethanol and suspended in 1× T4 DNA ligation buffer. The products of the previous step were equally divided and incubated at 16°C for 8 h with or without T4 DNA ligase. The reaction products were resolved by 15% denaturing PAGE in 0.5× TBE buffer. Gels were scanned by a fluorescent imaging system (Tanon 5200Multi, China). Moreover, we used 59 nt and 70 nt of 5′-strand FAM-labeled DNA oligonucleotides as Markers.

### Molecular docking analysis

The tertiary structure of SynRuvC, predicted by AlphaFold2, was downloaded from Uniprot (Q55506). The secondary structure of 5′-overhang was modeled by BIOVIA Discovery Studio Visualizer^2^, and modified using PyMOL. Docking simulations of SynRuvC and 5′-overhang was performed with *GRAMM*^3^ and the optimal binding mode was chosen based on the lowest docking energy (Majumder et al., 2012). PyMOL^4^ was used to display the three-dimensional figures and interactions of SynRuvC–5′-overhang (Laskowski and Swindells, 2011).

### Culture conditions

The cyanobacterium *Synechocystis* sp. PCC6803 was obtained from the Freshwater Algae Culture Collection of the Institute of Hydrobiology, Chinese Academy of Sciences. *Synechocystis* sp. PCC6803 strains were grown at 30°C in liquid BG-11 medium at pH 7.5 with 20 mM HEPES-NaOH on a rotary shaker (Chen et al., 2014). For growth on plates, 1.5% (weight/vol) agar were added to BG11. Cells were cultured at a light intensity of 40 μmol photons m^–2^ s^–1^ using cool-white fluorescent lamps. Mutant and overexpressed strains were maintained on solid BG11 agar plates supplemented with 50 μg/mL kanamycin. The optical density of the cells at 730 nm was measured to monitor growth.

### Construction of mutant and overexpressed strains

A conventional genetic approach was used to construct mutant and overexpression strains of *Synechocystis* sp. PCC6803 (Cameron and Pakrasi, 2010; Uchiyama et al., 2020). The *km^r^* gene replaced the coding sequences of *synruvC* (*sll0896*) through homologous recombination. Firstly, using appropriate primers, the upstream and downstream regions of the *sll0896* gene were amplified by PCR (Supplementary Table 1). Next, upstream and downstream PCR products were cloned into the pMD19T plasmid on either side of the *km^r^* gene to generate pMD19T-*sll0896*-delete. As shown in Supplementary Figure 10, the pMD19T-*sll0896*-overexpress plasmid was produced in the same way as the pMD19T-*sll0896*-delete plasmid. We used the strong bidirectional promoter *biPpsbA2* to overexpress *synruvC* gene expression (Zheng et al., 2023). Finally, wild type *Synechocystis* sp. PCC6803 was transformed using pMD19T-*sll0896*-delete and pMD19T-*sll0896*-overexpress to create mutant and overexpress SynRuvC strains, respectively. All mutant and overexpressed RuvC strains of *Synechocystis* sp. PCC6803 were identified by PCR, as shown in Supplementary Figure 10. Similarly, WT of *Synechocystis* sp. PCC6803 strains were also inserted the *km^r^* gene. Supplementary Table 1 lists all of the primers utilized in the study.

### RNA isolation and qRT-pCR analysis

The *Synechocystis* sp. PCC6803 strains were cultivated at 30°C in liquid BG-11 medium supplemented with 50 μg/mL kanamycin until reaching the mid-exponential growth phase. Subsequently, bacterial cells were harvested through centrifugation. Total RNA was isolated utilizing RNAprep Pure Cell/Bacteria Kit (Tiangen Biotech, Beijing, China) and treated with DNase I (Sigma-Aldrich). RNA purity and concentration were assessed using both gel electrophoresis and a spectrophotometer (NanoDrop, Thermo Scientific). And then, RNAs were reverse transcribed into cDNA using a Reverse Transcription kit (TransGen Biotech, Beijing, China) with random primers. Quantitative real-time PCR was performed with SYBR FAST qPCR Kit (Kapa Biosystems, USA) on a LightCycler^®^ 96 System (Roche), and the data are presented as the mRNA accumulation index (2-^ΔΔCt^). Data were normalized to WT strains (set as 1). *Rnpb* was used as the housekeeping gene (Zhang et al., 2009). All primers are listed in Supplementary Table 1.

### Growth under stress conditions

The strains of *Synechocystis* sp. PCC6803 (WT, *sll0896* knockdown mutant cells, and *sll0896* overexpressed cells) were cultivated to mid-logarithmic phase in BG11 (Sigma C#3061) medium supplemented with 50 μg/mL of kanamycin. Subsequently, the cultures were standardized to an OD_730_ = 1. Ten-fold serial dilutions were spotted onto fresh BG11 plates containing 50 μg/mL kanamycin and supplemented with or without 2 or 4 mM MMS (methyl methane sulfonate) (Beam et al., 2002; Nowosielska et al., 2006; Blanco et al., 2010). These plates were incubated at a light intensity of 40 μmol photons m^–2^ s^–1^ for 10 days at 30°C. Similarly, cyanobacterial growth curves were measured. All strains were cultured in liquid BG-11 medium containing 50 μg/mL kanamycin and supplemented with 2 mM MMS or 0.5 mM HU (Beam et al., 2002; Blanco et al., 2010), and the optical density was measured at 730 nm every day for 10 days. MMS and HU were purchased from Solarbio (Solarbio Life Sciences, Beijing, China). The experiments were performed in duplicate, at least three times.

### H_2_O_2_ tolerance assay

To test the tolerance of *Synechocystis* sp. PCC6803 strains to H_2_O_2_ stress, cells were subjected to increasing concentrations of H_2_O_2_ for 7 days (Sein-Echaluce et al., 2015). Cultures with an approximate OD_730_ of 1.5 were washed once with fresh BG-11 medium, and then 200 μL of the culture was dispensed into each well of a 96-well plate. H_2_O_2_ was supplemented to the wells at final concentrations of 0, 1, 2, 5, and 10 mM. The plate was incubated for 7 days in dark conditions at 30°C.

### Statistical analysis

Experimental data analyzed for significance were performed by using GraphPad Prism 6 (GraphPad Software, San Diego, California USA). Statistical analyses for the rest of the assays were performed using paired two-tailed Student’s *t*-test. Error bars represent ± SEM. *p < 0.05; **p < 0.01; ****p < 0.0001; n.s., not significant.

## Results

### Bioinformatics analysis of SynRuvC

A gene (*sll0896*) in the *Synechocystis* sp. PCC6803 genome is predicted to encode a RuvC homolog, and so was designated as *synruvC*. To compare SynRuvC with RuvCs from other bacterial taxa, the amino acid sequence of SynRuvC was aligned with those of RuvCs from *E*. *coli*, *P. aeruginosa*, *Mycobacterium leprae*, *Thermotoga maritima*, *Thermus thermophilus*, and *D. radiodurans* using the ESPript online server. As the details of conserved amino acid residues and secondary structure displayed in Supplementary Figure 1A, SynRuvC had >30% sequence identity with the RuvCs of other bacteria. Next, a phylogenetic tree of the RuvCs of a variety of species was generated using MEGA 6. Despite their high amino-acid sequence similarities, SynRuvC was genetically distant from those of other bacteria (Supplementary Figures 1A, B). The tertiary structures of SynRuvC dimer (predicted using Swiss-model), EcRuvC (PDB ID: 1HJR), and DrRuvC (PDB ID: 7W8D) were similar, suggesting functional conservation (Supplementary Figure 1C). Altogether, these results suggest that SynRuvC probably has conserved functions, but different properties compared to other RuvCs.

### Classical cleavage activity of SynRuvC

Recombinant SynRuvC was expressed in BL21(DE3) cells with a His-SUMO-tag fused to the N-terminus. The His-SUMO-tag was removed after elution of His-SUMO-SynRuvC from the Ni-NTA column, and after desalting purified SynRuvC was obtained by ion-exchange chromatography (HiTrap Q HP column) (Supplementary Figure 1D).

**FIGURE 1:**
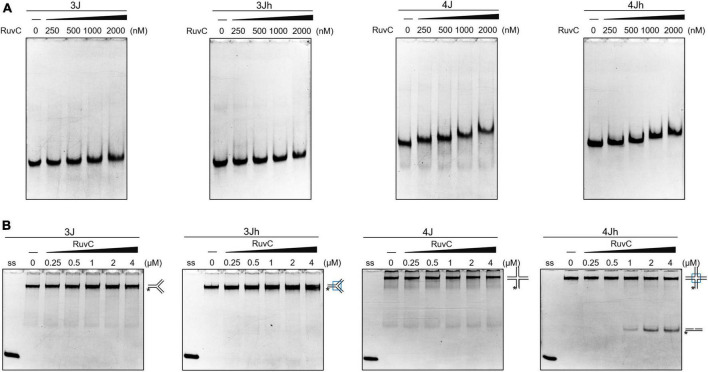
Classical cleavage activity of SynRuvC. **(A)** Binding activity analysis of SynRuvC with indicated DNA substrates using EMSA. The binding reactions contained 0, 250, 500, 1000, and 2000 nM of SynRuvC protein from left to right. All products were resolved by 5% native PAGE and fluorography. **(B)** Native PAGE analysis of SynRuvC cleavage of the indicated DNA structures. All products were resolved by 10% native PAGE and fluorography. ss: single-stranded DNA, *5′-FAM labeling in respective substrates.

HJ resolvase recognizes three- and four-way junctions in a structure-specific manner (Qin et al., 2022). The binding of SynRuvC to 3J (three-way junction with a non-homologous core), 3Jh (three-way junction with a 14 bp homologous core), 4J (four-way junction with a non-homologous core), and 4Jh (four-way junction with a 13 bp homologous core) (Supplementary Figure 2) was determined by electrophoresis mobility shift assay. SynRuvC slightly retarded the mobility of three- and four-way junctions and did not form stable complexes. SynRuvC bound to HJs with a variety of affinities (Figure 1A). However, SynRuvC specifically cleaved 4Jh, but not the other HJ substrates (Figure 1B). 4Jh has a 13 bp homologous core, allowing it to migrate within a certain range, whereas 4J is fixed and cannot migrate (Supplementary Figure 2). SynRuvC cleaved 4Jh but not 4J (Figure 1B), comparable to other RuvC homologs (Qin et al., 2022). However, there was no difference in the binding of these two types of four-way junctions by SynRuvC. Therefore, the homologous core of 4Jh affects the digesting activity, but not the binding activity, of SynRuvC.

**FIGURE 2:**
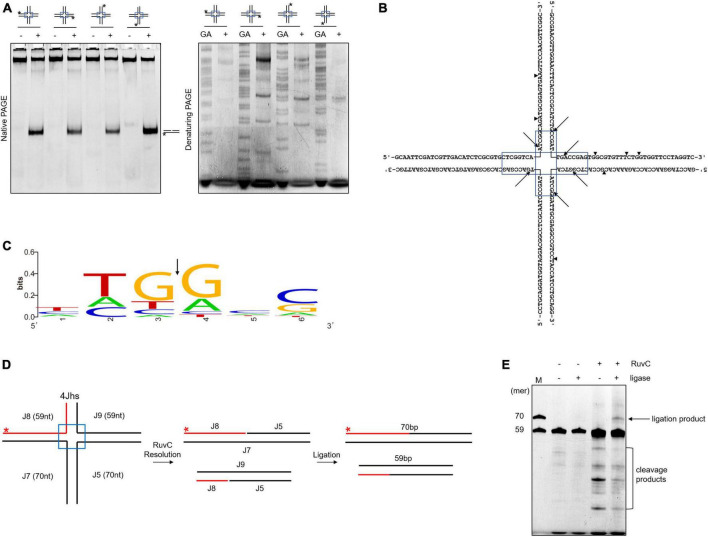
Cleavage sites determination and re-ligation of cleavage products in 4Jh. **(A)** 4Jh cleavage activity of SynRuvC. 4Jh, including one FAM-labeled at the 5′-end on the indicated strand of the four oligonucleotides, were employed as substrates. All products were resolved by 10% native PAGE or 15% denaturing PAGE, followed by fluorography. *Represents 5′-FAM labeling in respective substrates. FAM-labeled strands are on top, while + and - represent reactions with or without SynRuvC. GA denotes the Maxam-Gilbert GA ladders generated for each FAM-labeled strand. **(B)** The schematic of 4Jh cleavage sites by SynRuvC. Arrowheads indicate the locations of the cleavage. The boxed areas represented the mobile regions. **(C)** Conservation analysis of cleavage sites (from 16 cleavage site sequences). Black arrows indicate the cleavage position. **(D)** A schematic of an asymmetric 4Jh cleavage and re-ligation. 4Jhs, which contained the 5′-FAM-labeled short arm (59 nt in length) depicted with a red line, was resolved by SynRuvC to generate nicked duplex DNA. **(E)** Re-ligation assay of 4Jh cleaved by SynRuvC. The reaction was carried out as declared in Materials and Methods. Following the denaturing PAGE analysis, the products were observed by fluorography. Incubation with DNA ligase produced an extra band that co-migrated with the 70 nt marker. As markers (M), FAM-labeled oligos of prescribed length (70 and 59 nt) were utilized.

RuvC cleaves four-way junctions in a sequence-dependent manner, such as DrRuvC (5′-(G/C)TC↓(G/C)-3′), EcRuvC (5′-(A/T)TT↓(G/C)-3′), and PaRuvC (TTC) (Shah et al., 1994; Hu et al., 2020; Sun et al., 2022). To investigate whether SynRuvC cleaves in a sequence-dependent manner, the four oligonucleotide strand cleavage sites of 4Jh were analyzed. Surprisingly, 4Jh was cleaved at different sites on each oligonucleotide strand (Figures 2A, B). Additionally, some cleavage sites were not located in the homologous mobile region of 4Jh, similar to Hjc of *Pyrococcus furiosus* (Komori et al., 1999). The cleavage of 4Jh by SynRuvC exhibit no apparent sequence specificity. However, it is noticeable that the cleavage sites predominantly occur in the 5′-TG↓(G/A)-3′ sequence (Figure 2C).

To confirm that the nicked duplex DNA produced by SynRuvC can be ligated in a repair process similar to Hjc, we conducted an analysis using 4Jhs (4Jh with a labeled short strand) to examine the ligation products. After junction cleavage, the cleavage products of 4Jhs were treated with T4 DNA ligase, which converted them into a 70-nucleotides (nt) strand (Figures 2D, E). This suggests that nicked dsDNA is repaired by ligation. This indicated that SynRuvC cleaves 4Jhs symmetrically at related sites on both strands, leaving 5′-phosphate and 3′-hydroxyl termini.

### Nicked 4Jh cleavage activity of SynRuvC

Nicked HJs are involved in HR by acting as precursors for fully ligated HJ. Moreover, they are crucial intermediates in the generation of crossovers (Osman et al., 2003; Machin, 2020). To assess whether SynRuvC resolves these structures, we designed 12 possible nicked HJs (4Jhn) based on the 4Jh structure, where *n* is the position of the nick (nicked at position J2, J5, J6, or J7) and independently labeled the intact strands. The set of 12 substrates was incubated with SynRuvC as described in the *Materials and Methods*.

**FIGURE 3:**
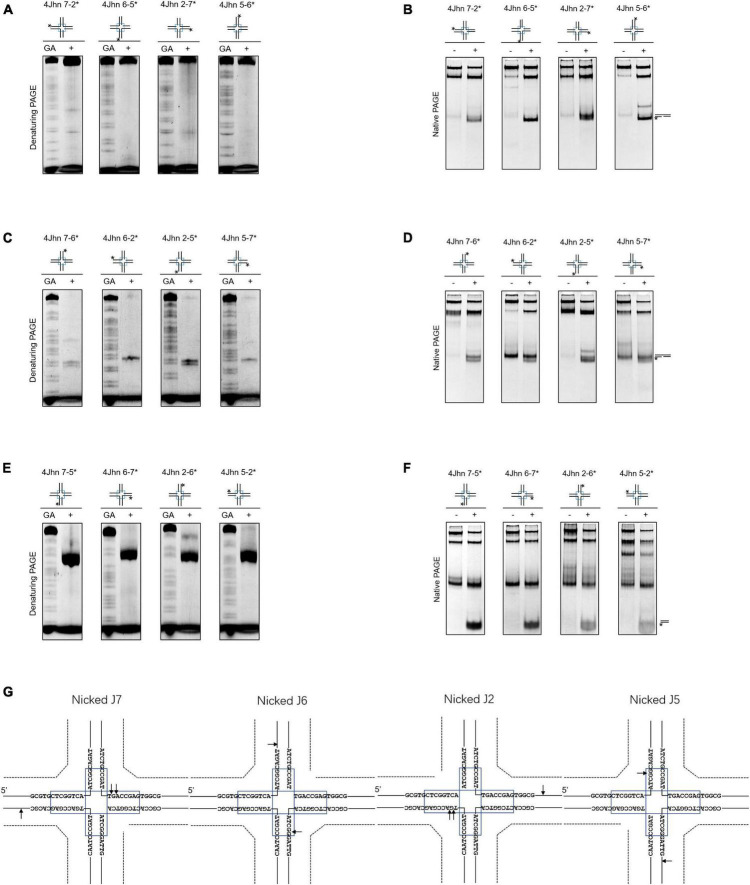
Nicked 4Jh cleavage activity of SynRuvC. The reactions of different nicked 4Jh and SynRuvC are the same as in Figure 2. **(A)** Reactions with nicked 4Jh substrates labeled on the strand opposite to the nick were resolved by 15% denaturing PAGE or **(B)** 10% native PAGE (cleavage products shown on the right). **(C,D)** Identical to panels **(A,B)**, but employing nicked 4Jh substrates labeled on the 3′-strand with respect to the nick. **(E,F)** Identical to panels **(A,B)**, but employing nicked 4Jh substrates labeled on the 5′-strand with respect to the nick. **(G)** Schematic representation of the main cleavage sites identified for the different nicked 4Jh substrates. Only the nucleotide sequence close to the branch point is given. Please take note of the discontinuity in one of the oligonucleotide chains. *Represents 5′-FAM labeling in respective substrates.

When the strand opposing the nick was labeled, no obvious cleavage by SynRuvC was observed in 4Jhn 7-2*, 4Jhn 6-5*, 4Jhn 2-7*, and 4Jhn 5-6* (Figure 3A). These four substrates were resolved by SynRuvC as determined by native PAGE (Figure 3B). These results suggested that these 4Jhn substrates may be cleaved on the other two strands, but not the labeled strands opposite the nick. As expected, when 4Jhn was labeled on the 5′- or 3′-strand with respect to the nick, cleavage by SynRuvC was detected (Figures 3C, E, G). Consistently, we detected nick or gap duplexes by native PAGE (Figure 3D). The cleavage sites within the homologous core predominantly had the pattern 5′-G↓(G/A)-3′ (Figure 3G).

Notably, the primary product of cleavage by SynRuvC of 4Jhn 7-5*, 4Jhn 6-7*, 4Jhn 2-6*, and 4Jhn 5-2* substrates was a short dsDNA (Figure 3F), indicating cleavage on the 5′-strand relative to the nick. Interestingly, the cleavage sites on the 4Jhn 7-5*, 4Jhn 6-7*, 4Jhn 2-6*, and 4Jhn 5-2* substrates were not perfectly symmetrical to the pre-existing nick in position 3′, resulting in products with overhangs and flaps (Figure 3G). The excision of one arm from a nicked HJ to form a three-arm structure has been reported for Yen1, AtGEN1, and AtSEND1 (Bauknecht and Kobbe, 2014), and is referred to as replication fork intermediate (Ref-I) cleavage activity (Supplementary Figure 3). Together, these results show that SynRuvC cleaves nicked 4Jh primarily by Ref-I and RF-like cleavage activities.

### Biochemical properties of the 4Jh resolvase activity of SynRuvC

Divalent metal ions are required as cofactors by HJ resolvases (Komori et al., 2000; Hu et al., 2020; Qin et al., 2022). Therefore, we used 4Jh as a substrate to investigate the metal-ion dependence of SynRuvC. We added MnCl_2_, MgCl_2_, ZnCl_2_, CaCl_2_, CuCl_2_, CoCl_2_, and NiCl_2_, to the reaction buffer and evaluated their effects on cleavage activity. Like DrRuvC, the activity of SynRuvC was significantly increased by MnCl_2_. Although MgCl_2_ could in part substitute for MnCl_2_, its stimulatory effect was considerably weaker. No activity was detected in the presence of ZnCl_2_, CaCl_2_, CuCl_2_, CoCl_2_, and NiCl_2_ (Supplementary Figure 4A). Mn^2+^ increased the cleavage activity of SynRuvC on 4Jh in a dose-dependent manner (Supplementary Figure 4B). At ≤10 mM Mg^2+^, the cleavage by SynRuvC of 4Jh increased in a dose-dependent manner (Supplementary Figure 4C). However, a high concentration of Mg^2+^ inhibited the cleavage activity of SynRuvC, consistent with a report on EcRuvC (Bennett et al., 1993).

To determine the optimum temperature, cleavage reactions were conducted at 4°C to 70°C. The activity of SynRuvC was enhanced at 37°C to 60°C but markedly reduced at >65°C (Supplementary Figure 4D). To further investigate its temperature stability, SynRuvC was pre-incubated at 30°C to 60°C for 15 min and incubated with 4Jh at 37°C for 1 h. SynRuvC activity was lost after incubation for 15 min at >55°C (Supplementary Figure 4E). Interestingly, SynRuvC showed considerable HJ cleavage activity at high temperatures such as 60°C but the pretreated protein was not active at 60°C. This might because that the protein quickly became inactivated within the 15-min timeframe (Supplementary Figures 4D, E).

Similar to PaRuvC (Hu et al., 2020), SynRuvC preferred alkaline conditions for 4Jh cleavage, with an optimum at pH 8.5–9.0 (Supplementary Figure 4F). SynRuvC was sensitive to the concentration of salt in the reaction mixture, with minor variations between K^+^ and Na^+^ (Supplementary Figure 4G). SynRuvC cleaved 4Jh in as little as 10 min, and the generation of cleavage products increased in a time-dependent manner for 60 min (Supplementary Figure 4H). Taken together, the optimum reaction conditions for the cleavage of 4Jh by SynRuvC is in a reaction buffer (20 mM Tris-HCl [pH 8.5], 50 mM NaCl, 1 mM DTT, 10% DMSO, 10 mM Mn^2+^, and 1% glycerol) at 50°C. To eliminate the possibility that SynRuvC failed to cleave 3J, 3Jh, and 4J because of unsuitable reaction conditions, these three substrates were evaluated under the optimal conditions. The results reaffirmed that SynRuvC remained incapable of cleaving any of these substrates (Supplementary Figure 5).

### Novel cleavage activity of SynRuvC in different DNA structures

**FIGURE 4:**
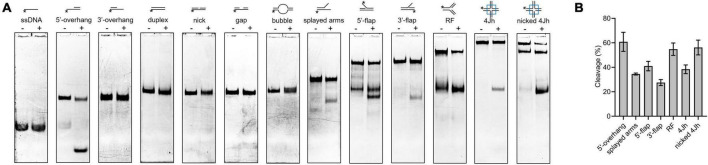
Substrate structure preference of SynRuvC. **(A)** Substrate specificity of SynRuvC nuclease activity. DNA substrates with 13 various structures (schematically illustrated on the uppermost) were prepared with a 5′-FAM labeled strand. 50 nM substrates were added with 10 mM Mn^2+^ treated with or without 2 μM SynRuvC at 37°C for 60 min. All products were resolved by 10% native PAGE, followed by fluorography. *Represents 5′-FAM labeling in respective substrates. **(B)** SynRuvC resolution efficiency toward various DNA structures. Normalized band intensity was quantified by ImageJ. Data are shown as mean ± SEM (*n* = 3).

The HJ resolvase, OsGEN-L, has 5′-flap endonuclease activity (Yang et al., 2012). To determine whether SynRuvC has novel activities other than conventional HJ recognition and cleavage, we evaluated its binding to (Supplementary Figure 6) and digestion of Figures 4A, B, ssDNA, 5′-overhang, 3′-overhang, duplex, nicked duplex, gapped duplex, bubble, splayed arms, 5′-flap, 3′-flap, and nicked 4Jh. The 5′-ends of the DNA structures were labeled with 6-FAM (Supplementary Table 1). SynRuvC cleaved 5′-overhang, splayed arms, 3′-flap, 5′-flap, and RF, in addition to classic HJs (Figure 4A). This indicated that SynRuvC exhibits robust FEN activity. Native PAGE showed that 5′-overhang was the optimum substrate for SynRuvC (Figure 4B). Also, SynRuvC primarily cleaved DNA substrates with 5′-arms. For instance, SynRuvC exhibited higher cleavage activities on DNA substrates with 5′- compared to 3′-overhang, as well as 5′- than 3′-flap (Figures 4A, B). Similar to DrRuvC, SynRuvC showed slightly stronger cleavage activity of pre-nicked 4Jh substrate compared to intact 4Jh (Figure 4B).

**FIGURE 5:**
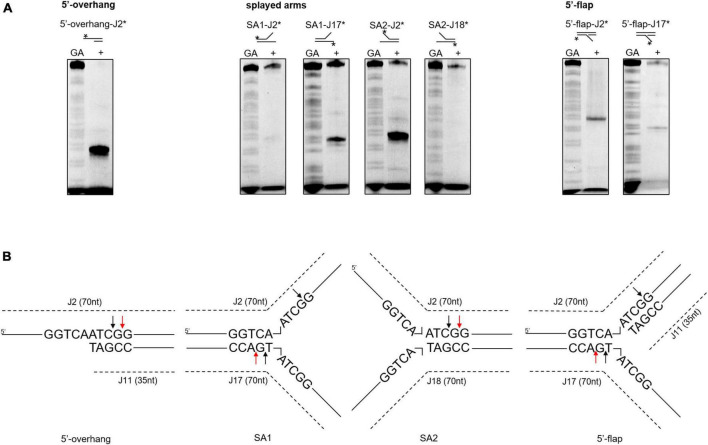
Mapping of SynRuvC incisions in different DNA structures. **(A)** Cleavage activities on SynRuvC in DNA substrates with a single strand. 50 nM of various DNA substrates 5′-end-labeled with FAM (asterisk) on each strand (marked on the top) and incubated with 2 μM SynRuvC at 37°C for 60 min, respectively. All products were resolved by 15% denaturing PAGE, followed by fluorography. **(B)** Schematic representation of cleavage sites identified in the 5′-overhang, splayed arms, and 5′-flap substrates. Only the nucleotide sequence close to the branch point is given. The arrow color shows favored cleavage sites for SynRuvC (red means main cleavage sites).

Next, we investigated whether the differences in cleavage activities were linked to binding affinities. According to a DNA binding assay, although all the DNA substrates were bound, SynRuvC preferred to bind DNA substrates with 5′-single strands than double strands (Supplementary Figure 6). Therefore, SynRuvC has a structural preference for DNA substrates. For high-resolution mapping of cleavage sites on branched DNA structures (5′-overhang, splayed arms, and 5′-flap), the reaction products were separated by 15% denaturing PAGE (Figure 5A). SynRuvC preferred to cleave the 5′-arms with a cleavage site on the 3′-strand of the duplex near the branch (Figure 5B) and showed a preference for cleavage sites with a 5′-G↓G/A-3′ pattern. These results showed that SynRuvC has FEN-like activity and cleaves a variety of non-HJ DNA substrates similarly to HJs.

### The binding and cleavage model of SynRuvC on 5*′*-overhang

**FIGURE 6:**
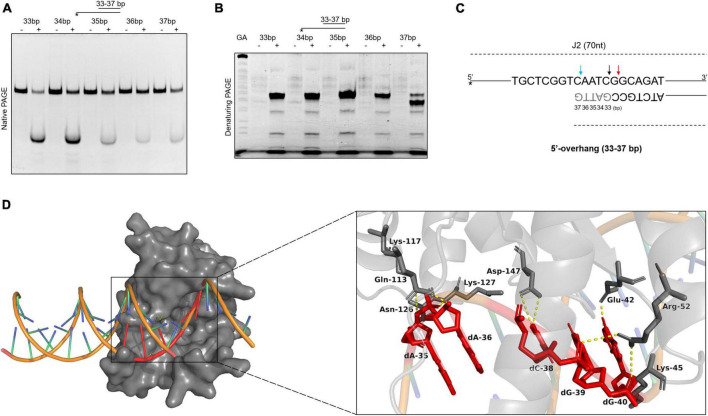
The binding and cleavage model of SynRuvC on 5′-overhang. **(A,B)** Cleavage activities on 5′-overhangs. DNA substrates with various lengths of 5′-overhang were prepared with 5′-strand FAM labeling (asterisk). 50 nM substrates were treated with or without 2 μM SynRuvC at 37°C for 60 min. All products were resolved by 10% native PAGE **(A)** and 15% denaturing PAGE **(B)**, followed by fluorography. **(C)** Schematic representation of cleavage sites identified for various lengths of 5′-overhang. Only the nucleotide sequence close to the branch point is given. The arrow color shows favored cleavage sites for SynRuvC. The major cleavage sites are shown by red arrows, whereas the secondary cleavage sites are indicated by black arrows. The color blue represents the new cleavage site generated when overhang was shorted. **(D)** Simulated binding model of SynRuvC and 5′-overhang. Docking simulations was performed with *GRAMM* and the three-dimensional figures were generated using PyMOL. Left: surface representation of the model of SynRuvC in complex with 5′-overhang (35 nt). 5′-overhang was shown as an orange DNA double helix. Right: schematic of the predicted interactions between SynRuvC and 5′-overhang. Potential hydrogen bonds were indicated as yellow dashed lines. All nucleotides represent the long chain of deoxyribonucleotides.

To further explore the binding and cleavage of SynRuvC to non-HJ DNA substrate, we used DNA substrates with 5′-overhangs of a variety of lengths. Surprisingly, the 5′-overhang cleavage activities varied with increasing overhang length, as determined by native PAGE (Figure 6A). To investigate the underlying mechanism, we evaluated the cleavage sites by 15% denaturing PAGE. The cleavage efficiency of 5′-CG↓GC-3′ decreased with decreasing length of 5′-overhang (30–40 bp) (Figure 6B and Supplementary Figure 7). A 5′-overhang of length 33 nt (opposite to the 37th on the complementary strand) generated a new cleavage site (5′-TC↓AA-3′) (Figures 6B, C and Supplementary Figure 7). These findings suggested that in addition to structural recognition, SynRuvC has a preferred cleavage sequence.

To evaluate the mechanism of SynRuvC binding and cleavage, we performed a docking simulation using the 3D structure of SynRuvC predicted by AlphaFold2 from Uniprot data and 5′-overhang (35 nt) predicted by BIOVIA Discovery Studio Visualizer. The optimum docking conformation, as determined by *GRAMM*, indicated that SynRuvC binds to the junction of the 5′-overhang (35 nt) at the single-double strand (Figure 6D). Deoxyribonucleotides dA-35, dA-36, dC-38, dG-39, and dG-40 of the 5′-overhang formed hydrogen bonds with the corresponding amino acid residues of SynRuvC (Figure 6D). The binding sites of Glu-42, Arg-52, Lys-117, and Lys-127 of SynRuvC with the 5′-overhang coincide with those reported for the interaction of RuvCs with HJs (Gorecka et al., 2013; Sun et al., 2022). These results indicated that after SynRuvC binds the single-double strand regions of 5′-overhangs of different lengths, it selects recognition and cleavage sites.

Subsequently, we designed a 3′-overhang and gapped duplex with a 33 nt overhang or 12 nt gap, distinct from the previous substrates, to investigate the cleavage activity of SynRuvC. The reaction conditions were as in Figure 4, and the products were resolved by 10% native or 15% denaturing PAGE (Supplementary Figure 8). SynRuvC cleaved both 3′-overhang-38 nt and gap-12 nt duplexes. In addition to its structural specificity, SynRuvC may also exhibit sequence specificity, necessitating the presence of an appropriate cleavage site in the binding region.

### Deletion of the C-terminus affects DNA cleavage by SynRuvC

To assess the function of the C-terminus in DNA binding and cleavage, we compared the binding and cleavage activities of the truncated protein SynRuvC^1–144^ and SynRuvC^FL^ *in vitro*. As the control, the highly conserved 75^th^ amino acid residue of SynRuvC (Supplementary Figure 1A), glutamic acid, was substituted with alanine. The mutant proteins were purified identically to SynRuvC^FL^ (Supplementary Figure 1D). DNA binding and cleavage reactions were carried out on 5′-FAM labeled 4Jh. The SynRuvC^E75A^ mutation in SynRuvC abolished its 4Jh binding and cleavage activities (Supplementary Figures 9A, B). Although deletion of the C-terminus slightly altered the migration of the protein-DNA complexes (Supplementary Figure 9A), it abolished the DNA cleavage activity of SynRuvC (Supplementary Figure 9B). A plausible explanation is that the amino acid D147 in α-helix 5 mediates substrate binding (Figure 6D). Hence, deletion of the C-terminus, which contains α-helix 5, resulted in the loss of SynRuvC^1–144^ cleavage activity. Therefore, deletion of the C-terminus hinders substrate binding, leading to loss of the cleavage activity of SynRuvC.

### SynRuvC is involved in tolerance to MMS, HU, and H_2_O_2_

**FIGURE 7:**
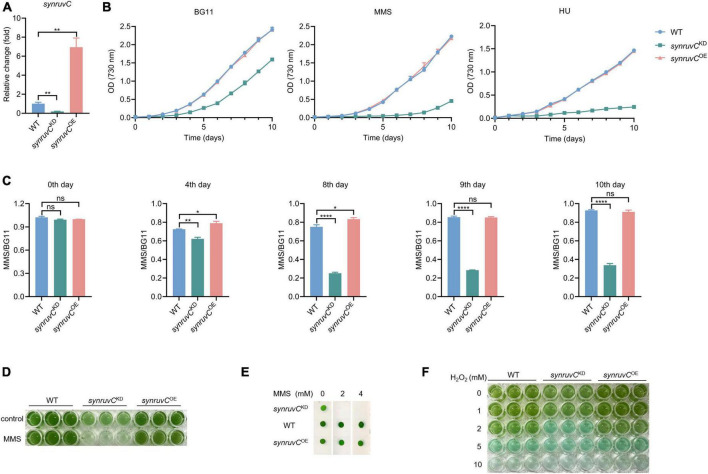
SynRuvC is involved in tolerance to MMS, HU, and H_2_O_2_. **(A)** Mutant strains examination using qRT-PCR. The gene expression of *synruvC* in WT, *synruvC^KD^*, and *synruvC*^OE^* Synechocystis* sp. PCC6803 strains were detected by qRT-PCR. **(B)** Growth of the WT, *synruvC^KD^*, and *synruvC^OE^* strains was assessed in BG11 medium with or without 2 mM MMS or 0.5 mM HU under standard culture conditions. The optical density was measured at 730 nm every day for 10 days. **(C)** The ratio of MMS to BG11 at OD_730_ for the WT, *synruvC^KD^*, and *synruvC^OE^* strains on various days of cultivation. **(D)** Growth of the WT, *synruvC^KD^*, and *synruvC^OE^* strains in BG11 or with 2 mM MMS. The culture and treatment conditions are the same as **(B)**. Cultures were plated into 96-well plates after 10 days of growth and photographed. **(E)** Phenotypic characterization of various strains treated with MMS. Ten-fold serial dilutions were spotted onto both fresh BG11 plates and BG11 supplemented with 2 or 4 mM of MMS. After 10 days of growth, plates were photographed. **(F)** Photograph of the 96-well plates containing WT, *synruvC^KD^*, and *synruvC^OE^* strains with increasing H_2_O_2_ concentrations. Data are shown as mean ± SEM (*n* = 3). *p < 0.05; **p < 0.01; ****p < 0.0001; n.s., not significant.

HR prevents methylation-induced toxicity in *E*. *coli* (Nowosielska et al., 2006). RadA, RecG, RecBCD, and RuvC, which function in HR, are involved in mitigating methylation-induced toxicity (Beam et al., 2002; Nowosielska et al., 2006). To gain insight into the physiological functions of SynRuvC, we employed conventional gene knockout and knock-in techniques for *Synechocystis* sp. PCC6803 (Cameron and Pakrasi, 2010; Uchiyama et al., 2020). We thus generated a strain overexpressing *synruvC*, designated *synruvC^OE^* (Supplementary Figures 10A, B). However, after several rounds of screening, we generated only *synruvC* knockdown strains (Figure 7A and Supplementary Figures 10C–E).

Photoautotrophic growth of *synruvC^KD^* in BG11 was slower than the WT and *synruvC^OE^* strains (Figure 7B). We analyzed growth of the WT, *synruvC^KD^*, and *synruvC^OE^* strains in BG11 medium supplemented with methyl methane sulfonate (MMS) or hydroxyurea (HU). Although growth of the WT and *synruvC^OE^* strains was inhibited by MMS or HU, the *synruvC^KD^* strain exhibited poorer proliferation than WT and *synruvC^OE^* strains (Figures 7B, C). Similarly, the WT and *synruvC^OE^* strains had a notable growth advantage in BG11 supplemented with or without MMS (Figures 7D, E). These results implicate SynRuvC in DNA repair in *Synechocystis* sp. PCC6803.

DNA repair proteins reverse the damage caused by the reactive oxygen species (ROS) (Stohl and Seifert, 2006) generated during photosynthesis. To investigate whether the expression level of SynRuvC affects tolerance to oxidative stress caused by exogenous hydrogen peroxide (H_2_O_2_) in *Synechocystis* sp. PCC6803 strains, 200 μL suspensions of the WT, *synruvC^KD^*, and *synruvC^OE^* strains were dispensed into wells of a 96-well plate containing H_2_O_2_. The *synruvC^KD^* strain was more sensitive to oxidative stress than the WT and *synruvC^OE^* strains (Figure 7F). Collectively, these results show that SynRuvC enhances tolerance to MMS, HU, and ROS.

## Discussion

HR facilitates genetic exchange and repairs DNA damage such as double-strand breaks and impediments at replication forks (West, 2003). RuvC proteins mediate the decomposition of HJ at the end of HR (van Gool et al., 1999; Donaldson et al., 2006). As a photoautotrophic organism, *Synechocystis* sp. PCC6803 requires robust DNA damage repair, particularly HR repair. In this study, we evaluated the biochemical properties, substrate structure, and sequence specificity of SynRuvC. To gain insight into the cleavage mechanisms of SynRuvC, we employed HJ and non-HJ structures as substrates for cleavage.

The model of canonical resolution dictates that symmetrical and coordinated incisions at HJs result in nicked DNA duplexes, which are sealed by ligases. Our findings show that SynRuvC has canonical resolvase activity. Symmetrical cleavage by SynRuvC of two sites on opposite strands of 4Jh generates a pair of nicked dsDNA (Figure 2A), similar to other HJ resolvases (Komori et al., 2000; Lilley and White, 2001). Furthermore, we ligated this nicked dsDNA *in vitro* using DNA ligase (Figure 2E). However, the Ref-I and RF-like cleavage activities of nicked 4Jh hampered assessment of the coordination of incisions (Figure 3 and Supplementary Figure 3). SynRuvC cleaves nicked 4Jh by arm-chopping, as do other HJ resolvases (Bauknecht and Kobbe, 2014; Carreira et al., 2022). However, the biological function of arm-chopping in the context of separating two DNA molecules linked by a junction is unclear. It has been hypothesized that the reversal of replication forks, resulting in the creation of a structure resembling a HJ, stabilizes stalled replisomes (Neelsen and Lopes, 2015). Moreover, if such intermediates persist during fission, arm-chopping by SynRuvC could facilitate their elimination.

SynRuvC cleaved non-HJ substrates, such as RF, flaps, and derivatives of flap structures (overhangs and splayed arms) (Figure 4A). This implies that, in addition to classical HJ resolution activity, SynRuvC has FEN activity. Moreover, SynRuvC exhibited a preference for binding to and cleaving DNA substrates with 5′-arms (Figure 4 and Supplementary Figure 6), distinguishing it from other RuvCs. Substrate recognition and cleavage by SynRuvC are similar to XPG proteins (Gen1 and Yen1) in terms of structural specificity and preference (Cloud et al., 1995; Yang et al., 2012; Carreira et al., 2022). These properties implicate SynRuvC in eliminating primer regions to facilitate the maturation of Okazaki fragments during lagging-strand synthesis, and multiple DNA repair (Lowder and Simmons, 2023).

The sequence specificity of junction cleavage is an important property of junction resolvases. Although the cleavage site of SynRuvC was primarily situated within the 5′-TG↓(G/A)-3′ sequence, SynRuvC did not show marked sequence specificity. Unlike EcRuvC, DrRuvC, and PaRuvC, which have cleavage sites of patterns 5′-(A/T)TT↓(G/C)-3′, 5′-(G/C)TC↓(G/C)-3′, and 5′-TTC-3′, respectively (Shah et al., 1994; Hu et al., 2020; Sun et al., 2022), SynRuvC had a broader range of cleavage sites. It was reported that Mn^2+^ reduced the sequence specificity of EcRuvC (Shah et al., 1994). SynRuvC showed a preference for the catalytic activity of Mn^2+^, which might contributed to its ability to cleave a broad range of sequences similar to EcRuvC. Notably, SynRuvC recognized a range of DNA sequences, facilitating repair of damaged DNA and enhancing its HJ resolvases activity in *Synechocystis* sp. PCC6803.

HR is a crucial cellular process for DNA replication, repair, chromosomes dynamics and cell development (Jasin and Rothstein, 2013; White et al., 2020; Spies et al., 2021). The resolution of HJ by resolvase is critical for the completement of HR (Kaczmarczyk et al., 2022). The HJ resolvases play important roles in cell development and stress resistance. For example, deficient of EcRuvC and the resulting failure of DNA repair disturb the cell growth and UV tolerance in *E.coli* (White et al., 2020; Bichara et al., 2021). In this study, we generated *synruvC* knockdown, but not knockout, strains after several rounds of screening (Figure 7A and Supplementary Figures 10C–E). This suggests *synruvC* to be an essential for *Synechocystis* sp. PCC6803. Knockdown of *synruvC* significantly inhibited the growth of *Synechocystis* sp. PCC6803. Initially, growth of the WT and *synruvC^OE^* strains was hindered by MMS. Subsequently, the self-repair mechanisms of these two strains resulted in increases in their growth rates to similar to those in BG11 (Figure 7C). Conversely, although the *synruvC^KD^* strain had a partially disrupted *synruvC*, it had reduced DNA repair and proliferation abilities. For instance, on day 10, growth of the WT strain resembled that in BG11. By contrast, the *synruvC^KD^* strain exhibited approximately three- to four-fold greater growth in BG11 compared to MMS (Figure 7C). Therefore, HJ resolvase SynRuvC is important for DNA repair and proliferation of *Synechocystis* sp. PCC6803.

In summary, we confirmed that SynRuvC has classic HJ resolvase activity and novel FEN activity *in vitro* and thoroughly described this activity, including its biochemical properties, structure specificity and sequence specificity. Moreover, SynRuvC has non-canonical arm-chopping (Ref-I and RF-like cleavage) activity for nicked 4Jh. Furthermore, by processing HJ and other abnormal DNA structures, *synruvC* maintains the genomic stability and viability of *Synechocystis* sp. PCC6803. Our findings substantiate the hypothesis that SynRuvC is involved in a variety of DNA repair mechanisms in *Synechocystis* sp. PCC6803.

## Data availability statement

The original contributions presented in this study are included in the article/Supplementary material, further inquiries can be directed to the corresponding authors.

## Author contributions

YG: Writing – review and editing, Writing – original draft, Validation, Methodology, Formal analysis, Data curation. YY: Writing – review and editing, Methodology, Funding acquisition, Conceptualization. CK: Writing – original draft, Investigation. YP: Writing – original draft, Investigation. WY: Writing – original draft, Investigation. JZ: Writing – original draft, Investigation. HJ: Writing – original draft, Investigation. XH: Writing – original draft, Investigation. YW: Writing – review and editing, Resources, Project administration, Funding acquisition, Conceptualization. XS: Writing – review and editing, Supervision, Resources, Project administration, Funding acquisition, Conceptualization.

## Footnotes

1
http://www.ncbi.nlm.nih.gov/

2
https://discover.3ds.com/

3
https://gramm.compbio.ku.edu/

4
http://www.pymol.org

## Supplementary material

Supplementary Figure 1 Bioinformatics analysis and purification of SynRuvC. **(A)** The sequence alignments of RuvC from different organisms were performed by the online server of ESPript. Secondary structural elements were illustrated above the sequences. The completely conserved amino acid residues were written in bold white characters and highlighted with a red background. Less conserved residues were written in bold black characters and highlighted with a yellow background. The asterisk indicated amino acid residues of the catalytic active center. **(B)** The phylogenetic tree of RuvC. The RuvC sequences from various species were aligned using ClustalW and the phylogenetic tree was generated by MEGA6. **(C)** The dimer tertiary structures of DrRuvC (PDB ID: 7W8D), EcRuvC (PDB ID: 1HJR), and SynRuvC (predicted using Swiss-model). **(D)** Purified proteins in this study. The purified SynRuvC^FL^ (Lane3), SynRuvC^E75A^ (Lane2), and SynRuvC^1–144^ (Lane1) proteins were subjected to 12% SDS-PAGE, followed by Coomassie Brilliant Blue staining.

Supplementary Figure 2 Structures and sequences of four types of DNA junctions. **(A)** 4J was prepared by annealing 4 DNA oligos with a non-homologous core sequence. **(B)** 4Jh was prepared by annealing 4 DNA oligos with a 13 bp homologous core sequence (blue rectangle). **(C)** 3J was prepared by annealing 3 DNA oligos with a non-homologous core sequence. **(D)** 3Jh was prepared by annealing 3 DNA oligos with a 14 bp homologous core sequence (blue rectangle).

Supplementary Figure 3 Schematic representation of the manners for the processing nicked 4Jh. Nicked 4Jh can be counter-nicked in the strand opposite the nicked one, yielding two nicked duplex molecules. Alternatively, the cleavage might happen on a strand adjacent to the nicked one, releasing a short duplex and an RF-like structure (Ref-I cleavage activity), which can be further incised into a short duplex and a nicked duplex molecule (RF-like cleavage).

Supplementary Figure 4 Biochemical characterization of SynRuvC in HJ cleavage activity. **(A–C)** Metal ion dependence: the HJ cleavage experiments were performed in the presence or absence of the indicated metal ions. Each metal ion was employed at 10 mM for the cleavage assay in panel **(A)**. **(D)** The effect of temperature on HJ cleavage activity. **(E)** Heat stability assay of SynRuvC. SynRuvC protein was preincubated at the indicated temperature for 15 min before the cleavage assay. **(F)** pH dependence: Tris–HCl was employed in the pH range of 6.5–9.0. **(G)** Effect of salts on HJ cleavage. NaCl or KCl concentrations varied as indicated. **(H)** Time-course analysis. Reactions were carried out at 37°C for different time. All products were resolved by 10% native PAGE and fluorography. Unless otherwise stated, 50 nM FAM-labeled 4Jh and SynRuvC protein (1 μM) were incubated in the reaction buffer for 60 min, as indicated in Materials and Methods. ss: single-stranded DNA. *5′-FAM labeling in respective substrates.

Supplementary Figure 5 The cleavage activity of substrate under optimal conditions. 50 nM FAM-labeled 3J **(A)**, 3Jh **(B)**, 4J **(C)**, and SynRuvC proteins (1 μM) were incubated in the pH 8.5 reaction buffer with 50 mM KCl and different doses of Mn^2+^ for 90 min at 45°C. Native PAGE analysis of SynRuvC cleavage of the indicated DNA structures. All products were resolved by 10% native PAGE and fluorography. ss: single-stranded DNA. *5′-FAM labeling in respective substrates.

Supplementary Figure 6 The DNA binding assays of SynRuvC toward different DNA structures. Bandshift analysis of the indicated DNA structures. The binding reactions contained 0, 250, 500, and 1000 nM of SynRuvC protein from left to right. All products were resolved by 5% native PAGE, followed by fluorography.

Supplementary Figure 7 Cleavage of 5′-overhangs with increasing lengths of duplex (30–40 bp). **(A)** Cleavage activities of SynRuvC. 5′-overhang DNA substrates were prepared with 5′-FAM labeled (asterisk) 70 nt strand with 11 short strands of different lengths (30–40 nt). 50 nM substrates were treated with 2 μM SynRuvC and 10 mM Mn^2+^ at 37°C for 60 min. **(B)** Negative controls of the cleavage reactions. All products were resolved by 15% denaturing PAGE, followed by fluorography.

Supplementary Figure 8 Other DNA structures can be cleaved after adjusting the sequence. **(A)** Analyzed the cleavage activity of SynRuvC on the 3′-overhang with a 38 nt overhang of 3′strand. **(B)** Analyzed the cleavage activity of SynRuvC on the gapped duplex with a 12 nt gap. The reactions are the same as in Figure 6, and all products were resolved by 10% native PAGE or 15% denaturing PAGE, followed by fluorography. *Represents 5′-FAM labeling in repsective substrates.

Supplementary Figure 9 Binding and cleavage analysis of WT and different SynRuvC mutations to the HJ. **(A)** HJ binding assay of SynRuvC^FL^, SynRuvC^E75A^, and SynRuvC^1–144^. 50 nM 4Jh was mixed with various concentrations of SynRuvC^FL^, SynRuvC^E75A^, or SynRuvC^1–144^ and incubated at 37°C for 60 min. All products were resolved by 5% native PAGE and fluorography. **(B)** HJ cleavage assay of SynRuvC^FL^, SynRuvC^E75A^, and SynRuvC^1–144^. 50 nM 4Jh was mixed with various concentrations of SynRuvC^FL^, SynRuvC^E75A^, or SynRuvC^1–144^ and 10 mM^2+^ and incubated at 37°C for 60 min. All products were resolved by 10% native PAGE and fluorography.

Supplementary Figure 10 Construction and PCR verification of the *sll0896* deletion mutants and overexpression strains. The left side is the schematic of the constructions of *sll0896* overexpression strains **(A)** or deletion mutants **(C)**. The gene of *sll0896* in the *Synechocystis* sp. PCC6803 chromosome was replaced with a light-inducible promoter inserted fragment *PpsbA*_2_-*sll0896*-*km^r^* **(A)** or a *km^r^* cassette **(C)**. The right panel of the ethidium bromide-stained agarose gel illustrated the overexpression **(B)** and the mutant **(D,E)** of *sll0896*. **(B)** Agarose gel electrophoresis was performed on PCR products (735 bp) amplified using *Ppsb*-seqF and *sll0896*R primers. **(D)** Agarose gel electrophoresis was performed on PCR products (1050 bp) amplified using *sll0895*F and *kan100*R primers. **(E)** Agarose gel electrophoresis was performed on PCR products (504 bp) amplified using *sll0896*F and *sll0896*R primers. WT, wild type strain; o1-7, difference overexpression strains; m1-7, difference mutant strains.
