# Supplementary figures and images for: Classical and novel properties of Holliday junction resolvase SynRuvC from Synechocystis sp. PCC6803
Source: Front Microbiol. 2024 Apr 18;15:1362880. doi: 10.3389/fmicb.2024.1362880 (PMC11063404; doi:10.3389/fmicb.2024.1362880)

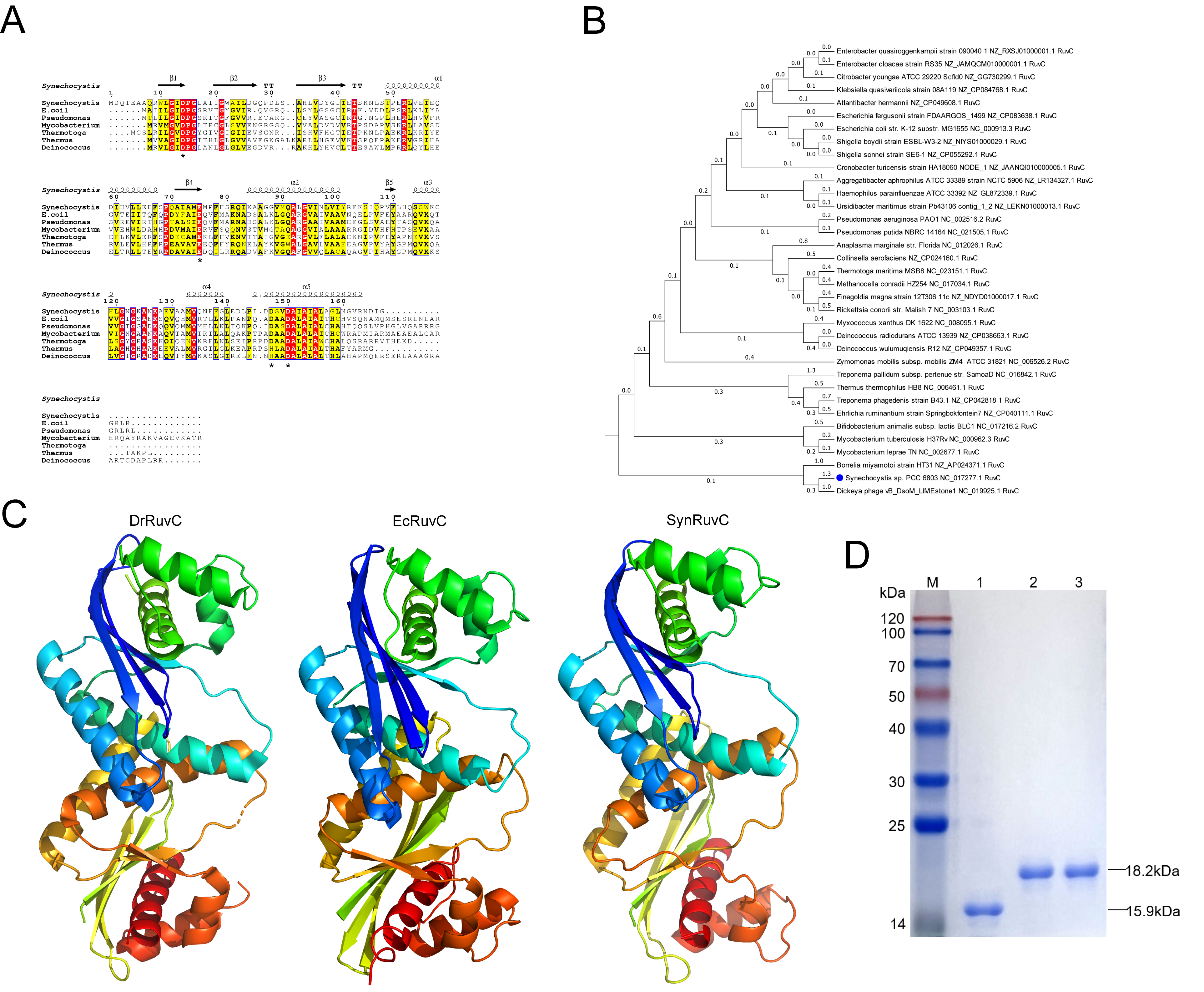

Supplement: Supplementary Figure 1 — Bioinformatics analysis and purification of SynRuvC. (A) The sequence alignments of RuvC from different organisms were performed by the online server of ESPript. Secondary structural elements were illustrated above the sequences. The completely conserved amino acid residues were written in bold white characters and highlighted with a red background. Less conserved residues were written in bold black characters and highlighted with a yellow background. The asterisk indicated amino acid residues of the catalytic active center. (B) The phylogenetic tree of RuvC. The RuvC sequences from various species were aligned using ClustalW and the phylogenetic tree was generated by MEGA6. (C) The dimer tertiary structures of DrRuvC (PDB ID: 7W8D), EcRuvC (PDB ID: 1HJR), and SynRuvC (predicted using Swiss-model). (D) Purified proteins in this study. The purified SynRuvCFL (Lane3), SynRuvCE75A (Lane2), and SynRuvC1–144 (Lane1) proteins were subjected to 12% SDS-PAGE, followed by Coomassie Brilliant Blue staining. [file Image_1.tif]

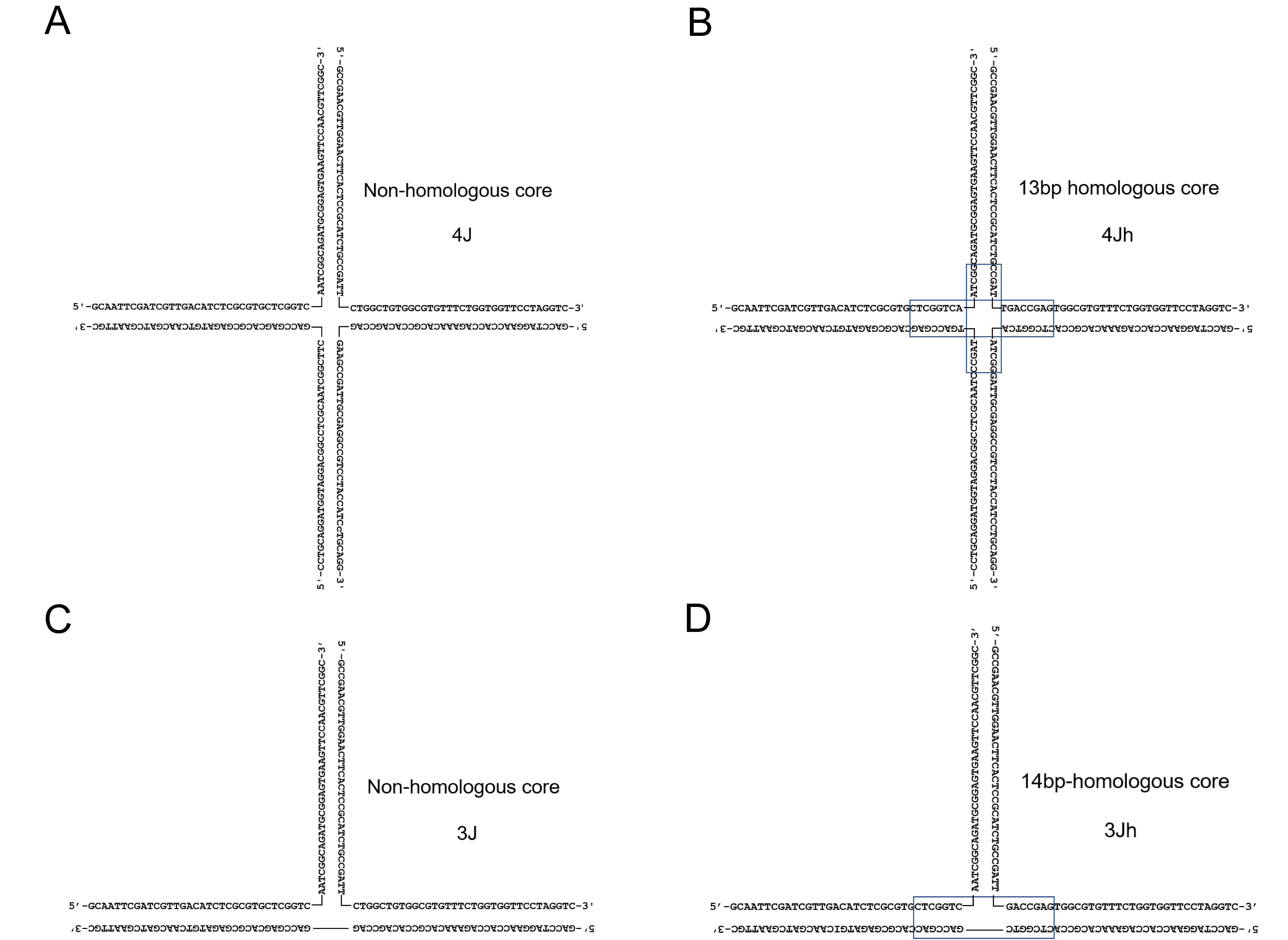

Supplement: Supplementary Figure 2 — Structures and sequences of four types of DNA junctions. (A) 4J was prepared by annealing 4 DNA oligos with a non-homologous core sequence. (B) 4Jh was prepared by annealing 4 DNA oligos with a 13 bp homologous core sequence (blue rectangle). (C) 3J was prepared by annealing 3 DNA oligos with a non-homologous core sequence. (D) 3Jh was prepared by annealing 3 DNA oligos with a 14 bp homologous core sequence (blue rectangle). [file Image_2.TIF]

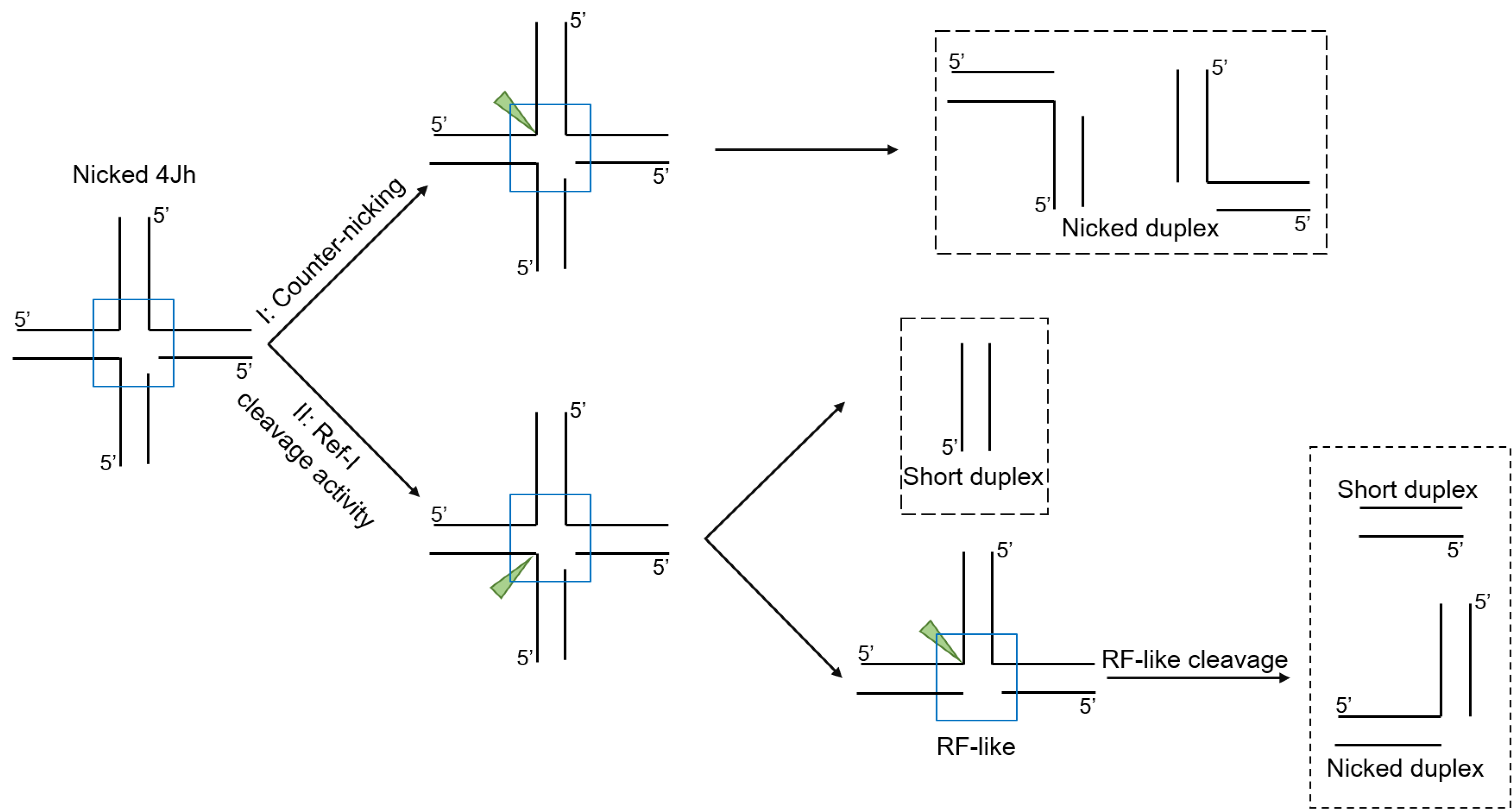

Supplement: Supplementary Figure 3 — Schematic representation of the manners for the processing nicked 4Jh. Nicked 4Jh can be counter-nicked in the strand opposite the nicked one, yielding two nicked duplex molecules. Alternatively, the cleavage might happen on a strand adjacent to the nicked one, releasing a short duplex and an RF-like structure (Ref-I cleavage activity), which can be further incised into a short duplex and a nicked duplex molecule (RF-like cleavage). [file Image_3.TIF]

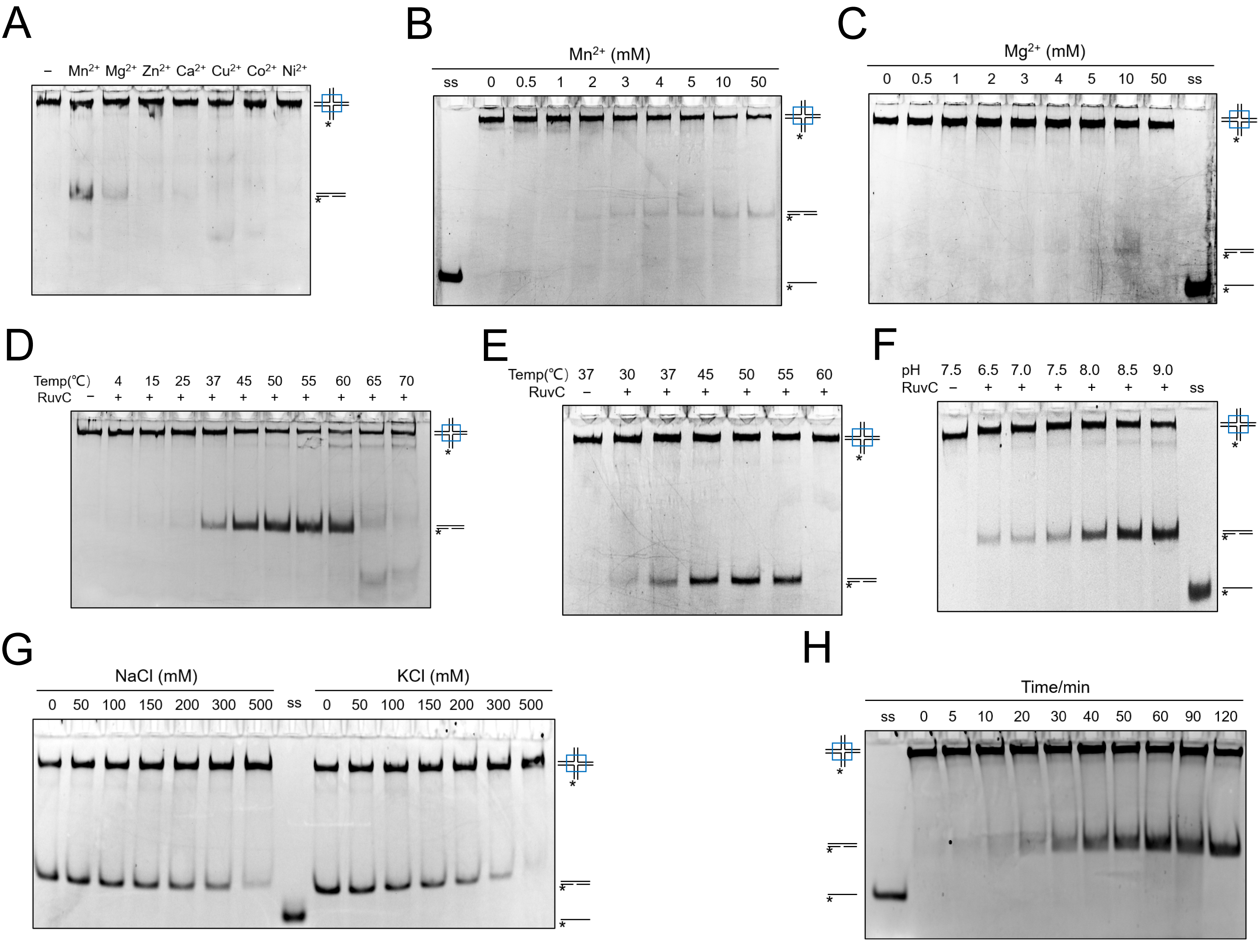

Supplement: Supplementary Figure 4 — Biochemical characterization of SynRuvC in HJ cleavage activity. (A–C) Metal ion dependence: the HJ cleavage experiments were performed in the presence or absence of the indicated metal ions. Each metal ion was employed at 10 mM for the cleavage assay in panel (A). (D) The effect of temperature on HJ cleavage activity. (E) Heat stability assay of SynRuvC. SynRuvC protein was preincubated at the indicated temperature for 15 min before the cleavage assay. (F) pH dependence: Tris–HCl was employed in the pH range of 6.5–9.0. (G) Effect of salts on HJ cleavage. NaCl or KCl concentrations varied as indicated. (H) Time-course analysis. Reactions were carried out at 37°C for different time. All products were resolved by 10% native PAGE and fluorography. Unless otherwise stated, 50 nM FAM-labeled 4Jh and SynRuvC protein (1 μM) were incubated in the reaction buffer for 60 min, as indicated in Materials and Methods. ss: single-stranded DNA. *5′-FAM labeling in respective substrates. [file Image_4.TIF]

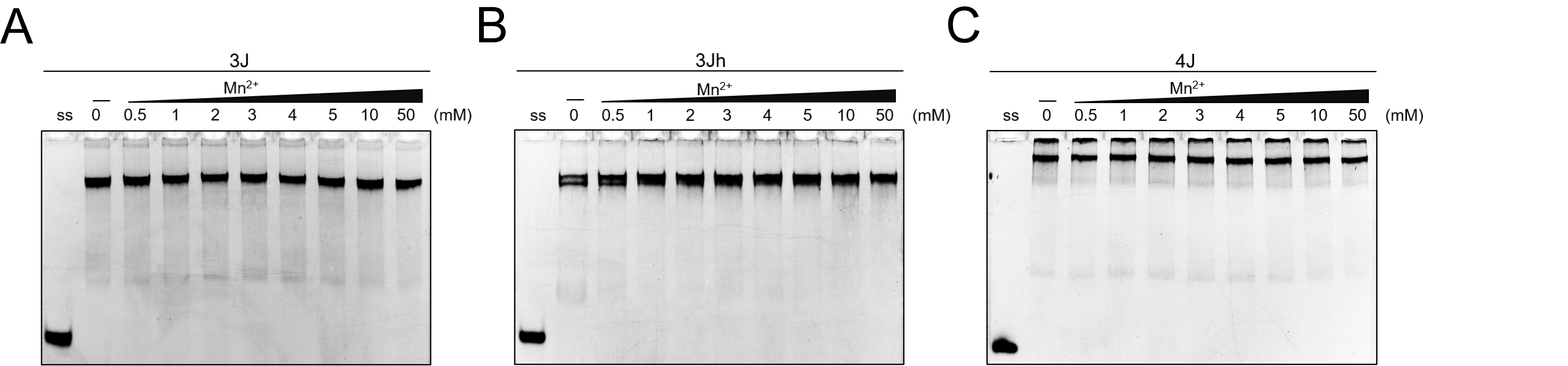

Supplement: Supplementary Figure 5 — The cleavage activity of substrate under optimal conditions. 50 nM FAM-labeled 3J (A), 3Jh (B), 4J (C), and SynRuvC proteins (1 μM) were incubated in the pH 8.5 reaction buffer with 50 mM KCl and different doses of Mn2+ for 90 min at 45°C. Native PAGE analysis of SynRuvC cleavage of the indicated DNA structures. All products were resolved by 10% native PAGE and fluorography. ss: single-stranded DNA. *5′-FAM labeling in respective substrates. [file Image_5.TIF]

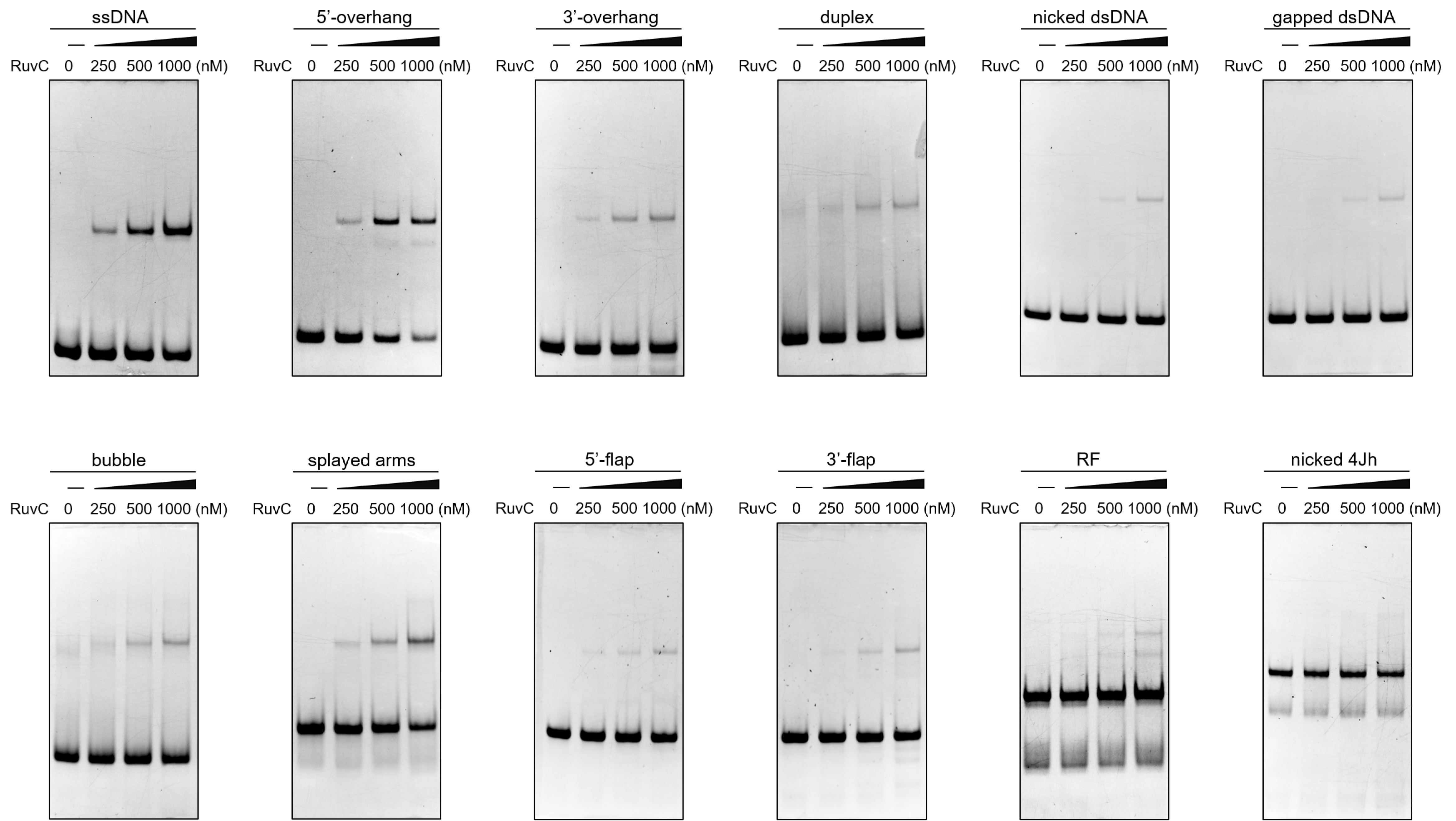

Supplement: Supplementary Figure 6 — The DNA binding assays of SynRuvC toward different DNA structures. Bandshift analysis of the indicated DNA structures. The binding reactions contained 0, 250, 500, and 1000 nM of SynRuvC protein from left to right. All products were resolved by 5% native PAGE, followed by fluorography. [file Image_6.TIF]

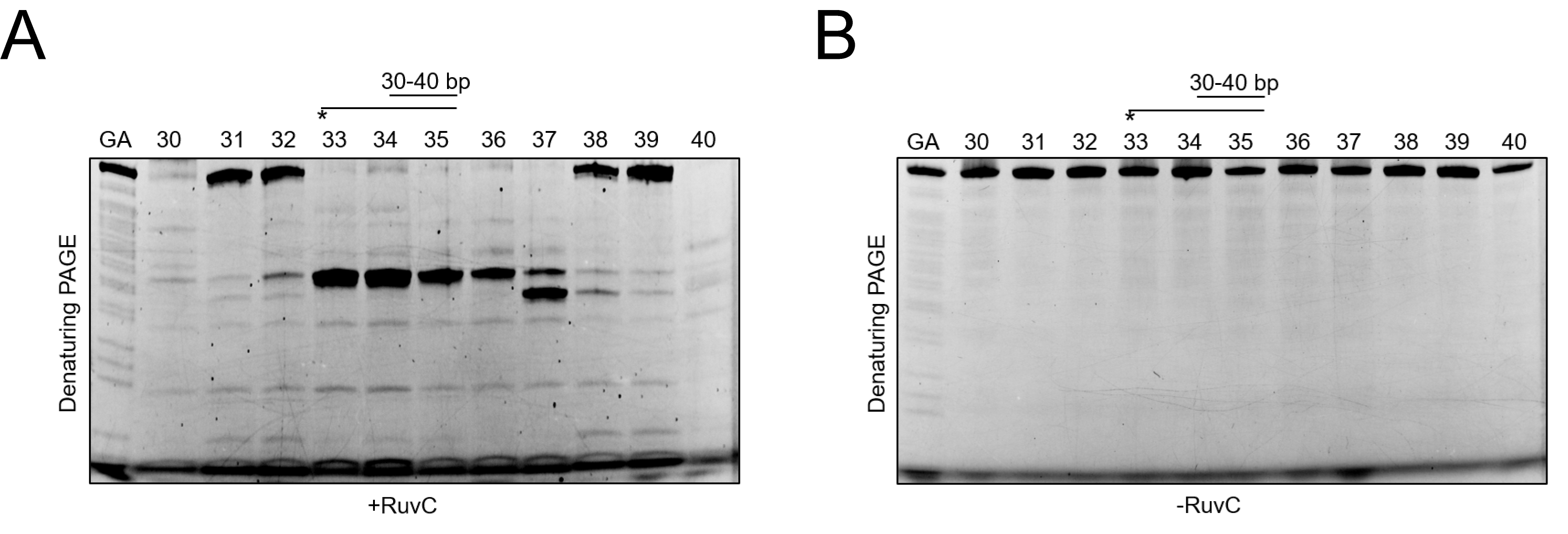

Supplement: Supplementary Figure 7 — Cleavage of 5′-overhangs with increasing lengths of duplex (30–40 bp). (A) Cleavage activities of SynRuvC. 5′-overhang DNA substrates were prepared with 5′-FAM labeled (asterisk) 70 nt strand with 11 short strands of different lengths (30–40 nt). 50 nM substrates were treated with 2 μM SynRuvC and 10 mM Mn2+ at 37°C for 60 min. (B) Negative controls of the cleavage reactions. All products were resolved by 15% denaturing PAGE, followed by fluorography. [file Image_7.TIF]

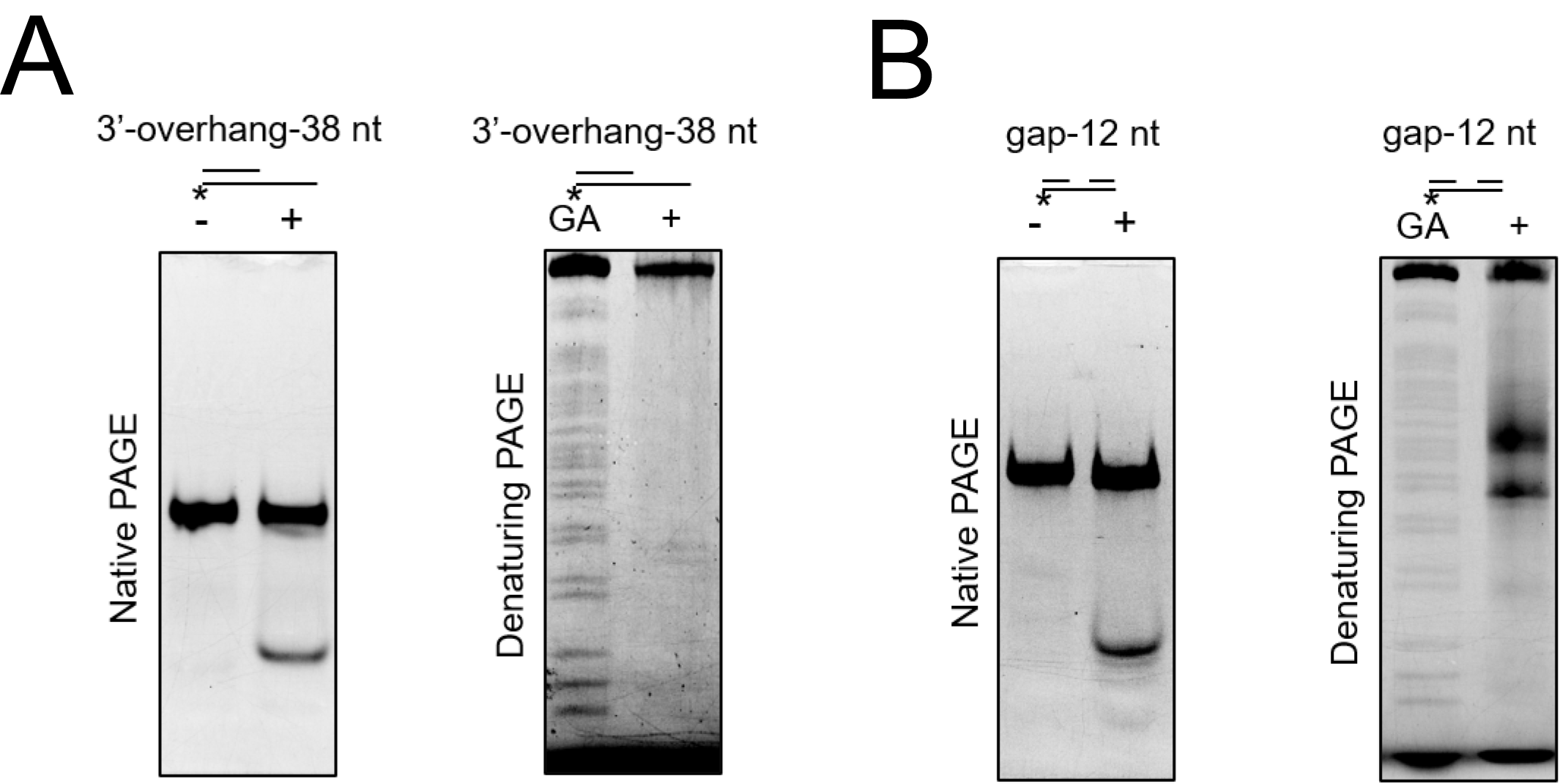

Supplement: Supplementary Figure 8 — Other DNA structures can be cleaved after adjusting the sequence. (A) Analyzed the cleavage activity of SynRuvC on the 3′-overhang with a 38 nt overhang of 3′strand. (B) Analyzed the cleavage activity of SynRuvC on the gapped duplex with a 12 nt gap. The reactions are the same as in Figure 6, and all products were resolved by 10% native PAGE or 15% denaturing PAGE, followed by fluorography. *Represents 5′-FAM labeling in repsective substrates. [file Image_8.TIF]

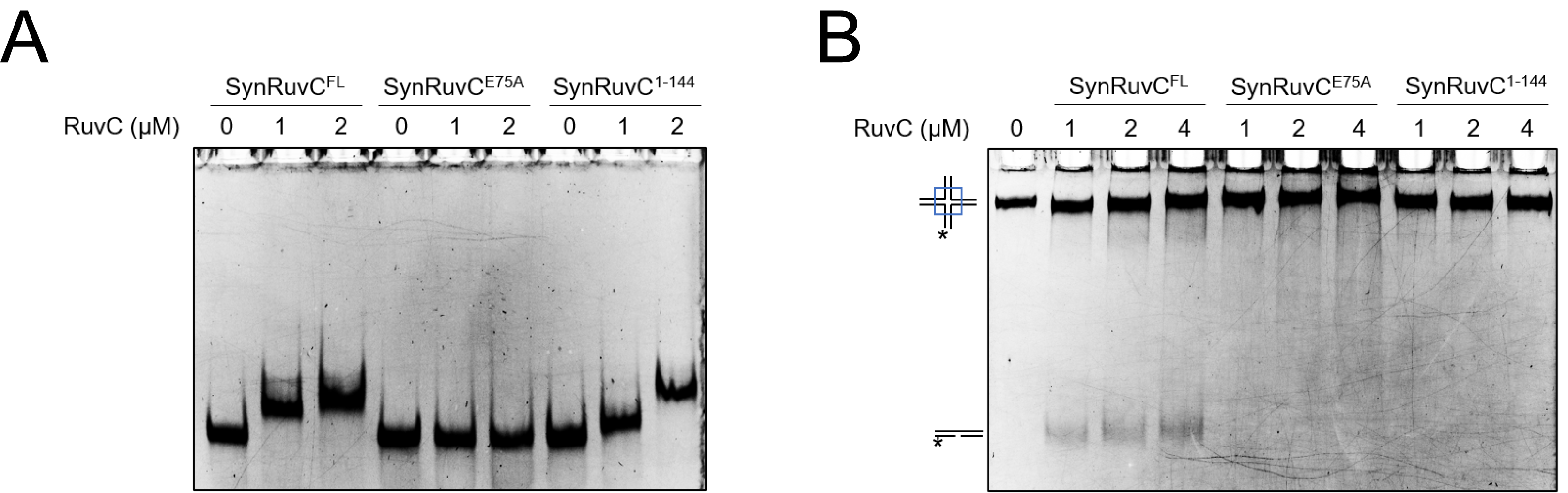

Supplement: Supplementary Figure 9 — Binding and cleavage analysis of WT and different SynRuvC mutations to the HJ. (A) HJ binding assay of SynRuvCFL, SynRuvCE75A, and SynRuvC1–144. 50 nM 4Jh was mixed with various concentrations of SynRuvCFL, SynRuvCE75A, or SynRuvC1–144 and incubated at 37°C for 60 min. All products were resolved by 5% native PAGE and fluorography. (B) HJ cleavage assay of SynRuvCFL, SynRuvCE75A, and SynRuvC1–144. 50 nM 4Jh was mixed with various concentrations of SynRuvCFL, SynRuvCE75A, or SynRuvC1–144 and 10 mM2+ and incubated at 37°C for 60 min. All products were resolved by 10% native PAGE and fluorography. [file Image_9.TIF]

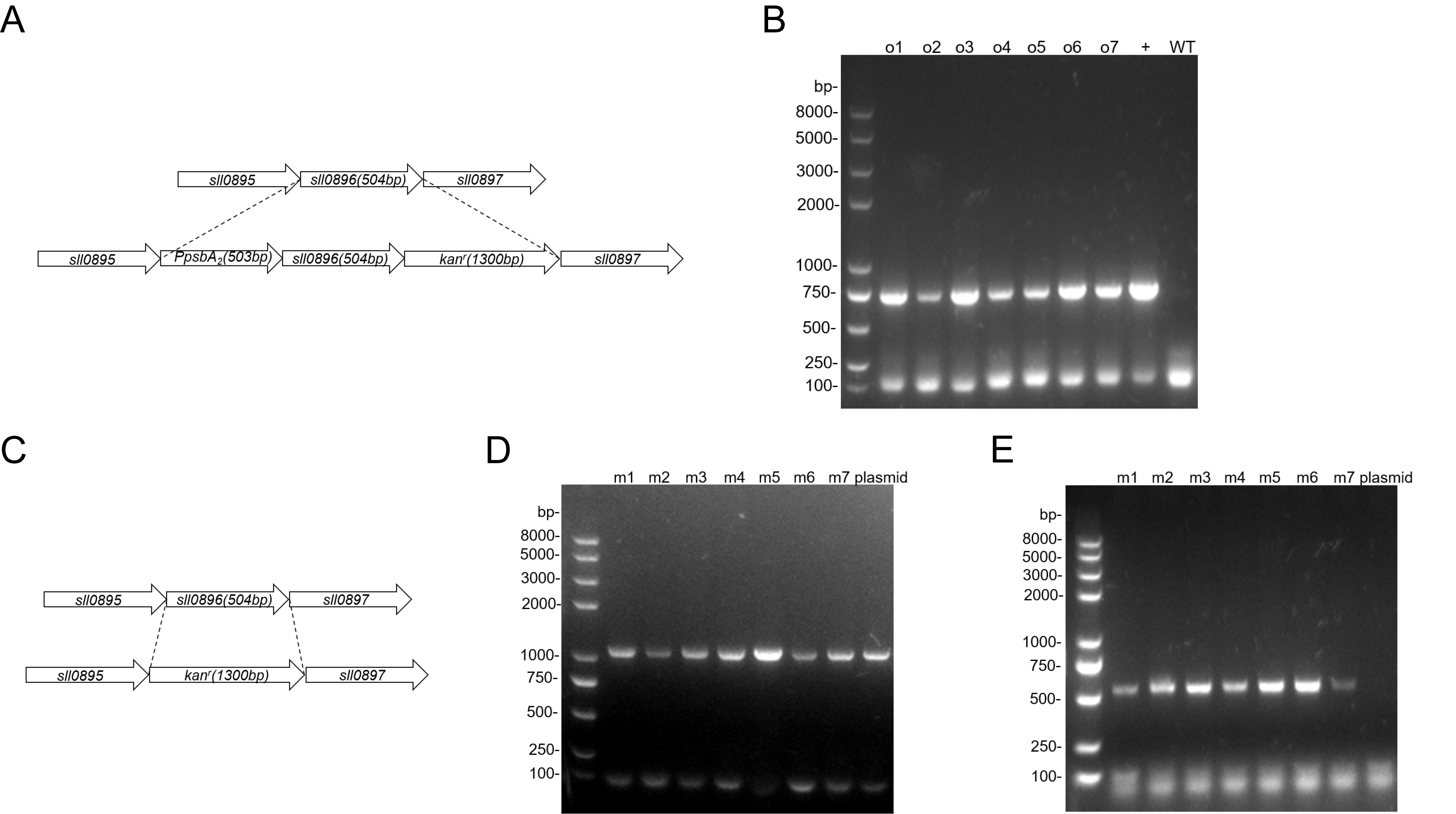

Supplement: Supplementary Figure 10 — Construction and PCR verification of the sll0896 deletion mutants and overexpression strains. The left side is the schematic of the constructions of sll0896 overexpression strains (A) or deletion mutants (C). The gene of sll0896 in the Synechocystis sp. PCC6803 chromosome was replaced with a light-inducible promoter inserted fragment PpsbA2-sll0896-kmr (A) or a kmr cassette (C). The right panel of the ethidium bromide-stained agarose gel illustrated the overexpression (B) and the mutant (D,E) of sll0896. (B) Agarose gel electrophoresis was performed on PCR products (735 bp) amplified using Ppsb-seqF and sll0896R primers. (D) Agarose gel electrophoresis was performed on PCR products (1050 bp) amplified using sll0895F and kan100R primers. (E) Agarose gel electrophoresis was performed on PCR products (504 bp) amplified using sll0896F and sll0896R primers. WT, wild type strain; o1-7, difference overexpression strains; m1-7, difference mutant strains. [file Image_10.TIF]
